# The wPDI Redox Cycle Coupled Conformational Change of the Repetitive Domain of the HMW-GS 1Dx5—A Computational Study

**DOI:** 10.3390/molecules25194393

**Published:** 2020-09-24

**Authors:** Jihui Gao, Peixuan Yu, Hongrui Liang, Jiahui Fu, Ziyue Luo, Dong Yang

**Affiliations:** Beijing Key Laboratory of Functional Food from Plant Resources, College of Food Science & Nutritional Engineering, China Agricultural University, 17 East Tsinghua Rd., Beijing 100083, China; jhgao@cau.edu.cn (J.G.); pxyu918@163.com (P.Y.); lhr_stem@cau.edu.cn (H.L.); s20193060969@cau.edu.cn (J.F.); gbwbya@163.com (Z.L.)

**Keywords:** wPDI, redox cycle, high molecular weight glutenin subunit, repetitive sequence, folding

## Abstract

The repetitive sequence of glutenin plays an important role in dough rheology; however, its interaction with wheat protein disulfide isomerase (wPDI) remains unclear. In this study, the conformations of wild type glutenin repetitive sequence (WRS) from the high molecular weight glutenin subunit (HMW-GS) 1Dx5, an artificially designed glutenin repetitive sequence (DRS) of which the amino acid composition is the same but the primary structure is different, and wPDI under different redox states were simulated. The molecular interactions between the aforementioned repetitive sequences with wPDI under different redox states were further investigated. The results indicated that the repetitive sequences bind to the b and b′ domains of an “open”, oxidized wPDI (wPDI^O^) which serves as the acceptor state of substrate. The repetitive sequence is partially folded (compressed) in wPDI^O^, and is further folded in the thermodynamically favored, subsequent conformational transition of wPDI^O^ to reduced wPDI (wPDI^R^). Compared with the artificially designed one, the naturally designed repetitive sequence is better recognized and more intensively folded by wPDI for its later unfold as the molecular basis of dough extension.

## 1. Introduction

The elastic gluten network formed by glutenin aggregation and crosslink endows dough extensibility [1,2,3]. Its network structure is mainly maintained by the intermolecular disulfide bonds formed between the N- and C-terminal domains of high molecular weight-glutenin subunit (HMW-GS) and non-covalent hydrogen bonding between the repetitive domains [4,5,6]. HMW-GS is categorized into x-type and y-type according to its molecular weight and amino acid sequences [7]. The intermediate repetitive domains of these two types of HWM-GS are mainly composed of 480–689 amino acid residues, and are rich in repeat sequences of proline, glutamine and glycine [8]. The repetitive domain in the y-type subunit is mainly composed of repetitive sequences of six amino acid residues (PGQGQQ) and nine amino acid residues (GYYPTSLQQ), while that in the x-type subunit is mainly composed of repetitive sequences of six (PGQGQQ), nine (GYYPTSPQQ) and three amino acid residues (GQQ) [9]. β-turn or γ-turn structures often form in the x-type and y-type repetitive domains, which further form a β-spiral structure similar to that of an elastic protein and promote the elasticity of dough [10,11,12]. In addition, the repetitive sequences of six amino acid residues are consistent in both the x and y subunits, but their proportions in these subunits are different. Glutamine in this six amino acid residue repetitive sequence is highly conserved; its abundance results in differences of glutenin conformational stability and its functional characteristics [13,14]. A higher content of glutamine residues in the repetitive sequence renders more likelihood of the formation of intramolecular and intermolecular hydrogen bonds. These hydrogen bonds break, reform and reach an equilibrium when external forces are applied and removed, displaying regions of “loop” and “train” which help the dough exhibit its elasticity [15].

Most of the flour improving reagents strengthen the gluten network through directly or indirectly oxidizing free sulfhydryl to form disulfide bonds, which in turn improve the rheological performance of dough and the gas retention property during the waking process. This ends up in the improvement of volume, homogeneity and texture properties of baking products [16,17]. However, flour improvers such as potassium bromate and azodicarbonamide are banned in most countries due to potential safety issues [18,19]. It is essential to study the crosslinking mechanism of the main backbone molecule of the gluten network and the catalysis mechanism of related enzymes. Wheat protein disulfide isomerase (wPDI) is a multifunctional enzyme located in the endoplasmic reticulum, which mediates the oxidation, reduction and isomerization of disulfide bonds and participates in the protein folding process [20]. Generally, PDI consists of four thioredoxin domains arranged in an a-b-b′-a′ manner, with a large number of acidic amino acid residues at its C-terminal [21]. Domains a and a′ contain the -CXXC- active sites that catalyze sulfhydryl/disulfide exchange reactions, while the non-catalytic domain b′ serves as the substrate binding site [22,23,24]. Additionally, PDI also exhibits another important function—a molecular chaperone, which promotes the folding of nascent or denatured proteins and restores their natural activities [25]. Many studies have shown that the addition of PDI effectively improves the alveographic properties of dough, mainly by catalyzing the formation of rheologically active disulfide bonds [26,27,28].

Currently, the mechanism of wPDI facilitated gluten network formation is limited to its catalyzed disulfide crosslinking in the N- and C-terminal domains of glutenin [27]. Neither the structure of wPDI nor how it interacts with the repetitive domain, which accounts for a larger proportion in glutenin, has been reported. Wheat flour products, such as the Chinese ramen and bread, expand extensively along one dimension or three dimensions during the baking or stretching process. However, little is known about the molecular basis of dough extension/expansion. While the comparatively short N- and C-terminals are mostly involved in the inter-molecular crosslinking, it is possible that the long repetitive domain is involved in the extension of dough. As one of the most significant HMW-GS, 1Dx5 is considered to play a key role in wheat flour farinograph properties, dough rheological and baking properties [29]. Therefore, this study focuses on the simulation of this player’s structures and their interactions under different redox states. This research offers further knowledge about how wPDI reacts with glutenin proteins that facilitate the formation of the gluten network in dough.

## 2. Results

### 2.1. Homology Comparison, Structure Simulation and Redox Conformations of wPDI

**Figure 1:**
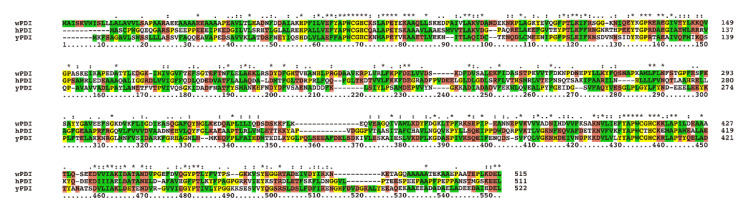
Amino acid sequence alignment of wheat, human, and yeast protein disulfide isomerase (PDI). “*” indicates identical amino acid residues, “꞉” indicates similar amino acid residues, and “ꞏ” indicates less similar amino acid residues. Yellow indicates amino acid residues G, P, C, H, Y; red indicates amino acid residues T, S, N, Q, E, D, K, R; green indicates amino acid residues W, L, V, I, F, A, M, C.

The primary sequences of wPDI and other PDIs with known three-dimensional structures, human (hPDI) and yeast (yPDI), were compared (Figure 1). wPDI shared 35% sequence homology with hPDI and 33% with yPDI, among which the -CGHC- sequences in the a and a’ domains were highly conserved. This highly conserved active site could enable wPDI to function in sulfhydryl/disulfide oxidation, reduction and isomerization. During the process of redox equivalent transfer to the substrate, the active site of -CGHC- is in dynamic equilibrium between the oxidation state (disulfide bond) and the reduction state (sulfhydryl group). The relative stability of these two forms determines the feasibility of the disulfide bond reduction in the active site. When the active site acts as an oxidant, the disulfide bonds are less stable and more difficult to form. Thus the disulfide is more easily transferred to the substrate. On the contrary, when the active site acts as a reductant, the disulfide bonds are relatively stable and more easily formed, rendering it more difficult to transfer to the substrate [21].

**Figure 2:**
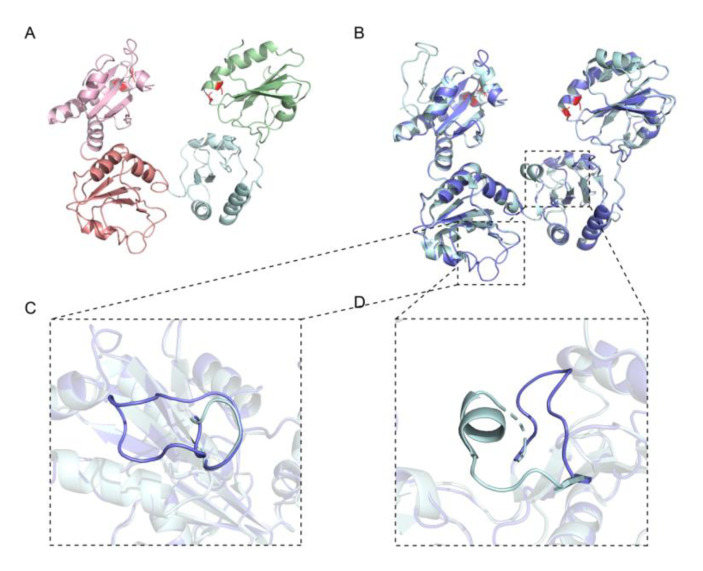
Simulated structure of wheat protein disulfide isomerase (wPDI). (**A**) the simulated structure of reduced wheat protein disulfide isomerase (wPDI^R^) with the template of human protein disulfide isomerase (hPDI) (reduced, PDB ID 4ekz). Domain a, b, b′ and a′ are colored as light pink, salmon, pale blue and pale green, respectively. (**B**) the structural superposition of wPDI^R^ with hPDI^R^. (**C**,**D**) the zoomed image of the extra loop structures in wPDI compared with hPDI.

The three-dimensional structures of wPDI under different redox states were modeled with that of its human counterpart (hPDI^R^ protein data bank (PDB) ID:4EKZ, hPDI^O^ PDB ID:4EL1) [30]. wPDI exhibited similar structures, in either its oxidized or reduced form, with that of its human counterpart, which contained four domains a, b, b′, and a′ (Figure 2A) [30]. There are subtle differences where there are two more loop structures (Loop1: P240-A265; Loop2: K293-A302) in wPDI (Figure 2B–D). It could be that these loop structures were inserted to represent the missing homologous sequence in hPDI.

**Figure 3:**
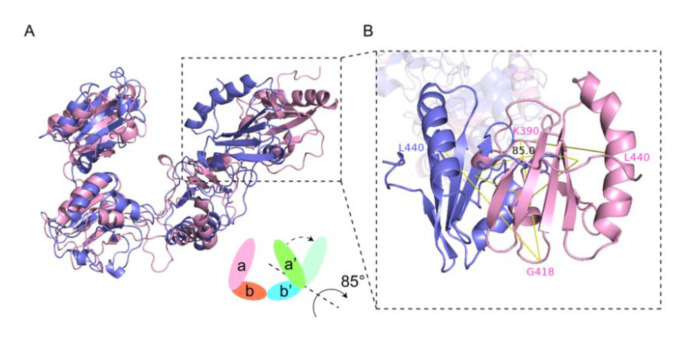
Conformation change of wPDI in its redox cycle. (**A**) the structural superposition of oxidized wheat protein disulfide isomerase (wPDI^O^) (pink) and wPDI^R^ (slate). The cartoon representation of the conformational change is below. (**B**) domain rotation of a′ during the redox cycle of wPDI. Taking G418 and K390 as the rotation axis, an 85° rotation takes place where the G418, K390, and L440 plane rotates from wPDI^O^ (light magenta) to wPDI^R^ (tv_blue).

To find out the conformational change of wPDI during its redox cycle, structures of reduced (wPDI^R^) and oxidized wPDI (wPDI^O^) were superimposed (Figure 3A). The root mean square deviation (RMSD) of the whole structure was 10.677 Å between these two, while the RMSDs of the a, b, b′ and a′ domains were 7.705 Å (V59-A170), 9.580 Å (S171-L285), 7.415 Å (L286-K390) and 16.190 Å (S391-D496), respectively. Although a, b, b′ domains remain largely the same during the redox transition of wPDI, their corresponding RMSD values are significant, indicating a local flexibility in its redox cycle. It is worth noting that the a′ domain takes a rotation of about 85° in the redox transition, which yields open and closed conformations for the oxidized and reduced state, respectively (Figure 3B). This suggested a highly flexible domain movement of wPDI in its redox cycle.

### 2.2. Structure Simulation and Comparison between Various Repetitive Sequences

**Figure 4:**
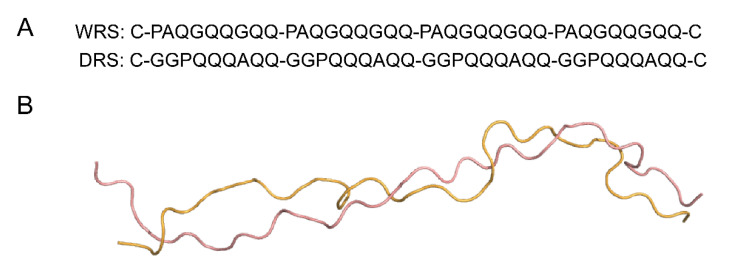
Primary and simulated tertiary structures of the repetitive sequences. (**A**) primary sequence of WRS (wild-type) and DRS (designed variant). (**B**) the simulated solution structures of free WRS (bright orange) and DRS (salmon).

The wild type repetitive sequence (WRS) of HWM-GS 1Dx5 containing four repeating units (PAQGQQGQQ×4) and a designed repetitive sequence (DRS) with identical amino acid compositions but different primary sequences (GGPQQQAQQ×4) were studied here (Figure 4A). The solution conformations of WRS and DRS were simulated, respectively (Figure 4B). First of all, both WRS and DRS exhibited a disordered loop conformation in solution while WRS presented a slightly tighter conformation than DRS. The distance between the N- and C-terminals in WRS is 79.9 Å while that in DRS is 85.1 Å. Meanwhile, hydrogen bonding was formed in both WRS and DRS, resulting in γ/β-turn structures, which is consistent as previously reported (Figures S1 and S2) [11,12].

### 2.3. Interactions between WRS and wPDI^O/R^

**Figure 5:**
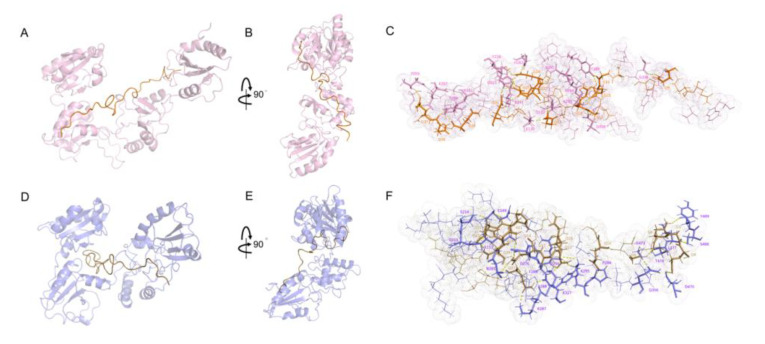
Interaction between WRS and wPDI^O/R^. (**A**,**B**) interaction between WRS and wPDI^O^ cartoon representations of wPDI^O^ (pink) and WRS (orange). (**C**) stick representations of amino acid residues involved in the interaction between wPDI^O^ (pink) and WRS (orange). (**D**,**E**) interaction between WRS and wPDI^R^ cartoon representations of wPDI^R^ (slate) and WRS (sand). (**F**) stick representations of amino acid residues involved in the interaction between wPDI^R^ (slate) and WRS (sand). Dotted amino acid residues are involved in the hydrophobic interactions and the dotted line indicates hydrogen bonds.

Generally, WRS interacts with wPDI^O^ at its b and b′ domains where WRS inserts into the plane (defined by the four domains of wPDI^O^) with an angle of approximately 45° (Figure 5A,B). WRS is well embedded in the wPDI^O^ groove and its N-terminal is on the back side of wPDI^O^ (plane) and close to its a′ domain while the C-terminal is on the front side of wPDI^O^ (plane) and close to its b domain (Figure 5A,B and Figure S3A). To quantify the structural changes of wPDI^O^ after binding with WRS, the RMSD between apo-wPDI^O^ and wPDI^O^ in the WRS–wPDI^O^ complex was calculated to be 3.135 Å, suggesting little change of the wPDI^O^ after its binding with WRS.

Interestingly, when wPDI^O^ transited to its reduced state—wPDI^R^, the binding pattern of WRS to wPDI^R^ is vastly different. At this point, the N-terminal of WRS is on the front side of wPDI^R^ (plane) and close to its a′ domain while the C-terminal extends to the back of wPDI^R^ (plane) and close to its b domain (Figure 5D,E and Figure S3A). After binding with WRS, wPDI^R^ exhibited an RMSD of 3.583 Å compared with its apo-form. Moreover, the reduction of wPDI not only led to the change of WRS binding pattern, but also resulted in the compression of WRS as discussed in Section 2.5. During the binding of WRS to b and b′ domains of wPDI^O/R^, the interactions between WRS and L212, D215, K216, V273, H282, L286, Y288, P289, N292, A293, P294, K295 and A335 are conserved.

Free in solution, WRS forms 11 hydrogen bonds with 9 γ/β-turn structures. Under the action of wPDI^O^, WRS forms 15 hydrogen bonds and 8 γ/β-turn structures. Once wPDI^O^ gets reduced to wPDI^R^, it forms 20 hydrogen bonds and 5 γ/β-turn structures. After binding to wPDI^O^ and then wPDI^R^, the number of γ/β-turn structures decreased and the hydrogen bonds formed outside γ/β-turn structure increased (Supplementary Materials Figure S1). The CDOCKER energy is related to the binding energy of the protein–ligand complex. The lower the energy value, the more thermodynamically favored complex formed [31]. The CDOCKER energy during WRS binding to wPDI^O^ is −316.7 kJ/mol. There are 27 hydrogen bonds formed between WRS and wPDI^O^ and 65 amino acid residues were involved in their hydrophobic interactions. About 90% of the amino acid residues in b and b′ domains are involved in the interaction (Figure 5C and Figure S4). On the other hand, the CDOCKER energy during WRS binding to wPDI^R^ is −351.7 kJ/mol. There are 27 hydrogen bonds formed between them, and 66 amino acid residues are involved in their hydrophobic interactions. The proportion of amino acid residues involved in the interaction between b and b′ domains is about 60%, as shown in Figure 5F and Figure S4. During the transition of oxidized to reduced wPDI, the CDOCKER energy got lower and residues involved in hydrophobic interactions got higher. This indicated binding complex between WRS and wPDI^R^ is more thermodynamically favored and the WRS–wPDI^O^ to WRS–wPDI^R^ transition happens spontaneously.

### 2.4. Interactions between DRS and wPDI^O/R^

**Figure 6:**
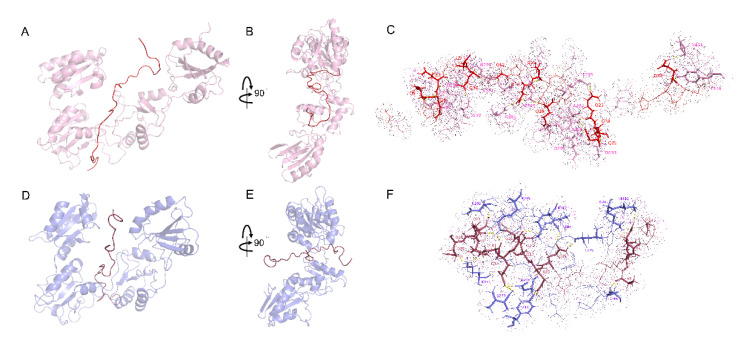
Interaction between DRS and wPDI^O/R^. (**A**,**B**) interaction between DRS and wPDI^O^; cartoon representations of wPDI^O^ (pink) and DRS (tv_red). (**C**) stick representations of amino acid residues involved in the interaction between wPDI^O^ (pink) and DRS (red). (**D**,**E**) interaction between WRS and wPDI^R^ cartoon representations of wPDI^R^ (slate) and DRS (raspberry). (**F**) stick representations of amino acid residues involved in the interaction between wPDI^R^ (slate) and DRS (raspberry). Dotted amino acid residues are involved in the hydrophobic interactions and the dotted line indicates hydrogen bonds.

Vastly different from WRS, binding of DRS to wPDI almost happens within the wPDI (plane). Bound to wPDI^O^, the N-terminal of DRS is inserted through the space between b and b′ domains on the front of wPDI^O^ while the C-terminal is close to its a′ domain (Figure 6A,B and Figure S3B). The RMSD between apo-wPDI^O^ and itself in the complex is 3.017 Å. Once wPDI transits from its oxidative state to reductive state, DRS binds within wPDI^R^ with its N-terminal domain, being inserted through the b and b′ domains on the back of the wPDI^R^ (plane). Meanwhile the C-terminal of DRS is in the middle of the a and a′ domains (Figure 6D,E and Figure S3B). wPDI^R^ exhibited an RMSD of 3.214 Å after binding with DRS. In addition, there is also a compression of DRS during the redox transition of wPDI (see Section 2.5 for details). I264, K277, Y288, P289, Q290, N292, A293, and K295 are conserved in the DRS interaction with wPDI^O/R^ (Figure 6C,F).

Free in solution, DRS forms eight hydrogen bonds with 7 γ/β-turn structures. Bound to wPDI^O^, DRS forms 4 hydrogen bonds with 4 γ/β-turn structures, and 25 hydrogen bonds with 13 γ/β-turn structures once bound to wPDI^R^. Binding with wPDI^O^ decreased the γ/β-turn structures and hydrogen bonds formed within DRS. When wPDI is reduced, both the γ/β-turn structures and hydrogen bonds within DRS increase and exceed those in the free solution state (Figures S2 and S4).

The CDOCKER energy during DRS binding to wPDI^O^ is −309.444 kJ/mol during which a total of 18 hydrogen bonds are formed, and 61 amino acid residues are involved in the hydrophobic interactions. About 80% amino acid residues in b and b′ domains are involved in the interaction (Figure 6C and Figure S4). On the other hand, the CDOCKER energy during DRS binding to wPDI^R^ is −329.228 kJ/mol. A total of 17 hydrogen bonds are formed and 45 amino acid residues are involved in the formation of hydrophobic interactions between DRS and wPDI^R^. In the b and b′ domains of wPDI^R^, about 95% amino acid residues are involved in their interactions (Figure 6F and Figure S4). Justified by CDOCKER energy, DRS also forms a more stable complex with wPDI^R^.

During the redox cycle of wPDI, the b and b′ domains of wPDI^O/R^ bind WRS and DRS at different sites. For wPDI^O^, K277, Y288, P289, N292, A293, P294, K295, D325 and I355 are conserved for binding both WRS and DRS. However, there are 25 and 20 different amino acid residues only involved in binding to WRS or DRS, respectively. For wPDI^R^, K187, Y216, D276, L286, K287, Y288, F289, N290, N292, A293, K295, K327 are involved in the interactions with both WRS and DRS. There are 10 and 11 different amino acid residues only involved in binding to WRS or DRS, respectively.

### 2.5. wPDI-Mediated Folding of the Glutenin Repetitive Sequence

**Figure 7:**
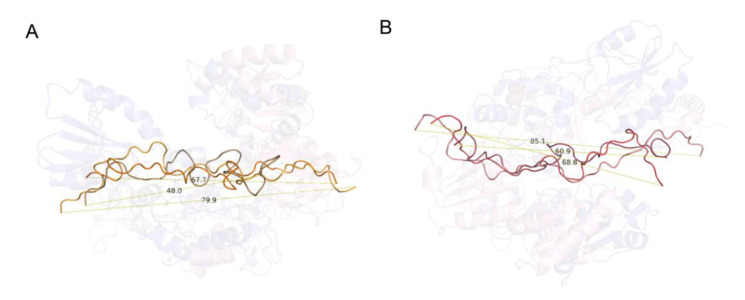
Compression of the repetitive sequence by wPDI. (**A**) cartoon representation of WRS free in solution (bright orange), bound with wPDI^O^ (orange) and wPDI^R^ (sand). (**B**) cartoon representation of DRS free in solution (salmon), bound with wPDI^O^ (tv_red) and wPDI^R^ (raspberry). The repetitive sequences are aligned and corresponding wPDI are transparent in the background. The distance between the C_α_ at the N- and C-terminals of the repetitive sequences were measured.

As mentioned above, both WRS and DRS are compressed upon binding to wPDI^O^, and the compression further develops once wPDI^O^ is reduced to wPDI^R^. Straightforwardly, the distance between the N-and C-terminals of glutenin repetitive sequence decreases step by step as the wPDI redox cycling goes on. Binding to wPDI^O^ compressed WRS from 79.9 Å to 67.7 Å, and it is further compressed to 48.0 Å while the reduction of wPDI takes place (Figure 7A). DRS is also compressed from 85.1 Å to 68.8 Å in the initial binding step and then to 60.9 Å in the redox cycle (Figure 7B). In the entire process, WRS is compressed by 31.9 Å while DRS is compressed by 24.2 Å. Such compression provides the basis for the future extension of the gluten network under the action of external forces.

## 3. Discussion

Studies have shown that hPDI contains multiple binding sites with affinities ranging from medium to low [32,33,34]. These binding sites are conserved to a certain extent and bind with substrates of different sizes, shapes or folding degrees. It also helps to release the substrate from the PDI when the reaction is complete. Due to its high structural similarity with hPDI and the highly conserved a, b, b′, a′ domains, wPDI is able to recognize and bind with WRS and DRS with different patterns. The extra loops in wPDI may help to stabilize wPDI since wheat is cultivated in a more stressed environment, which will be further illustrated with experimental evidence.

In the redox cycle of hPDI, its conformational change mainly occurs in the b′xa′ region [35]. Similar to hPDI, the a′ domain in wPDI undergoes a rotation of about 85° during the reduced to oxidized state transition that leads to a more open conformation. This is consistent with the conclusion that hPDI exposes its hydrophobic amino acid residues in the a′ and b′ domains during the reduced to oxidized transition to facilitate substrate binding [20]. It was found that the number of amino acid residues in the b and b′ domain of wPDI^O^ that interact with WRS is significantly higher than that in wPDI^R^, suggesting that wPDI^O^ could be the substrate acceptor state. Initial binding of the HMW-GS repetitive domain to wPDI^O^ brings about the first compression of this region.

During the transition from wPDI^O^ to wPDI^R^, the CDOCKER energy decreased for both the WRS and DRS, indicating that a thermodynamically favored complex was formed during the reduction of wPDI. In our study, only the repetitive sequence was involved thus no disulfide was transferred to the HWM-GS. However, this could be the scenario in reality that reduction of wPDI brings about the oxidation/crosslinking of the N- and C-terminals of glutenins. The number of amino acid residues involved in the interaction between WRS and b-b′ domains of wPDI decreased during the reduction of wPDI. This could be due to the further compression/folding of the repetitive domain which resulted in less exposure of amino acid residues involved in the interaction between b and b′ domains. Evacuation of the substrate binding sites prepares the release of WRS and the recognition of the next, less folded one. The structural differences, as indicated by calculated RMSDs, are much larger between oxidized and reduced wPDI than those between repetitive sequence bound- and apo-wPDI. This suggests that the wPDI conformational change induced by the redox cycle is more significant than that induced by substrate binding.

To investigate the difference between nature designed and artificially designed repetitive sequences, DRS was applied to the same simulation in parallel with WRS. In general, wPDI interacts more strongly with WRS than with DRS. For both wPDI^O^ and wPDI^R^, WRS interacts with wPDI^O/R^ with more hydrogen bonds and hydrophobic interactions, lower CDOCKER energy, more hydrogen bonds formed within itself, and more extensive compression. This indicates that the nature designed repetitive sequence in the HMW-GS is better recognized by wPDI and able to fold more extensively for the subsequent unfolding in dough.

## 4. Materials and Methods

### 4.1. Homology Comparison

The wheat (GenBank: AAA19660.1), human (GenBank: AAC50401.1) and yeast (GenBank: BAA00723.1) PDI sequences were searched in the GenBank^TM^ database, and homologous comparison was conducted with the Clustal.X software [36].

### 4.2. Structural Simulation of wPDI and Glutenin Repetitive Sequence

The Swiss model (https://swissmodel.expasy.org) website was employed to simulate the wPDI structures at various redox state with hPDI at its reduction state (PDB: 4EKZ) and oxidation state (PDB: 4EL1) as templates, respectively [37]. The initial structure of the glutenin repetitive sequence (WRS, DRS) was generated with PyMol [38]. Molecular dynamics simulation was carried out to optimize each above molecule’s solution structure and the details of MD simulation are contained in the following paragraph.

### 4.3. MD Simulation of the Solution Structures and Interaction between wPDI^O/R^ and the Repetitive Sequences

Briefly, the aforementioned structures of wPDI^O^, wPDI^R^, WRS, and DRS were subjected to MD simulations, respectively, for solution structure optimization. Then, molecular docking between different repetitive sequences and wPDI redox states (WRS to wPDI^O^, WRS to wPDI^R^, DRS to wPDI^O^, DRS to wPDI^R^) was performed with the DS CDOCKER module. Among the 10 wPDI-substrate binding conformations generated, the complex with lowest CDOCKER energy was subjected to MD simulation again for their final solution states.

The BIOVIA Discovery Studio software V16.1.0 was employed to perform the MD simulations based on the CHARMm36 molecular mechanics and molecular dynamics force field engine. In the DS standard dynamics cascade, molecular dynamics was performed. All systems were solvated in an approximately 60 × 82 × 116 Å^3^ orthorhombic box with a minimum clearance of 7 Å using the explicit periodic boundary water model and neutralized by the addition of sodium cation and chloride anion to an ionic concentration of 0.08. Initially, the system underwent two energy minimization steps: 1000 steps of steepest descent minimization and 2000 steps of conjugate gradient minimization with the adopted Newton–Paphson algorithm [39]. The following three steps of heating, equilibration, and production were performed afterwards. The whole system was heated from 50 K to 298.15 K in 4 ps without constraints, then the equilibrium step was run at 298.15 K for 20 ps without constraints. The following production step was run at 298.15 K and pressure of 1.0 for 200 ps with typed NPT and no constraints. The electrostatic parameter was set to automatic which recognizes the periodic environment and used the Particle Mesh Ewald (PME) electrostatic calculation. Among the 100 conformations generated, the one with the lowest total energy was selected for the subsequent study.

For wPDI-substrate interaction analysis, LigPlot was used to analyze the hydrophobic interactions and PyMol was used to analyze the hydrogen bonds [40].

## 5. Conclusions

The highly conserved amino acid residues in wPDI impart it the possibility to recognize the repetitive domains of HMW-GS. The repetitive sequence is compressed upon binding to wPDI^O^ and further folded by the subsequent conformational change during the redox transition of wPDI^O^ to wPDI^R^. Compared with the artificially designed one, the naturally designed repetitive sequence is better recognized and folded by wPDI for the later unfolding as the molecular basis of dough extension.

## Supplementary Materials

The Supplementary Materials are available online. Figure S1: The γ/β-turn structure of WRS in three states. Figure S2: The γ/β-turn structure of DRS in three states. Figure S3: Conformational change of repetitive sequence in the redox cycle of wPDI. Figure S4: Statistics of interactions between repetitive sequence and wPDI.
