# Supplementary material for: The wPDI Redox Cycle Coupled Conformational Change of the Repetitive Domain of the HMW-GS 1Dx5—A Computational Study
Source: Molecules. 2020 Sep 24;25(19):4393. doi: 10.3390/molecules25194393 (PMC7583805; doi:10.3390/molecules25194393)
Supplement: Supplementary file 1 [file molecules-25-04393-s001.pdf]

Supplementary Materials: The wPDI redox cycle coupled conformational change of the repetitive domain of the HMW-GS 1Dx5-A computational study

Jihui Gao †, Peixuan Yu †, Hongrui Liang †, Jiahui Fu, Ziyue Luo and Dong Yang *

Beijing Key Laboratory of Functional Food from Plant Resources, College of Food Science & Nutritional Engineering, China Agricultural University, 17 East Tsinghua Rd., Beijing 100083, China; jhgao@cau.edu.cn (J.G.); pxyu918@163.com (P.Y.); lhr_stem@cau.edu.cn (H.L.); s20193060969@cau.edu.cn (J.F.); gbwbya@163.com (Z.L.)

* Correspondence: dyang@cau.edu.cn

† These authors contributed equally to this work.

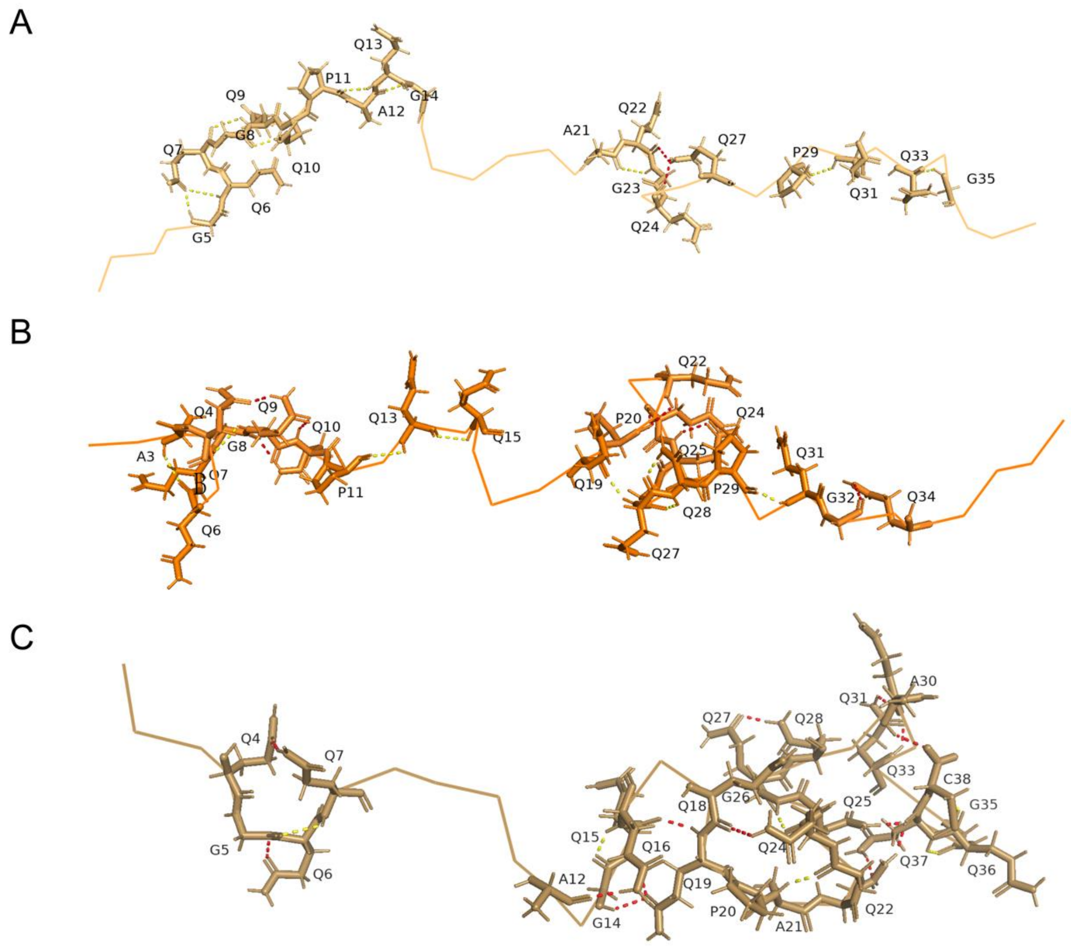

Figure S1. The γ/β-turn structure of WRS in three states. Ribbon representation of the γ/β-turn structure of WRS free in solution (A, brightorange), bound with $wPDI^{O}$ (B, orange) and $wPDI^{R}$ (C, sand). Amino acids involved in structural maintaining hydrogen bonding are shown in sticks. Yellow dotted line represents hydrogen bonds involved in the γ/β-turn structures and red dotted line represents hydrogen bonds not involved.

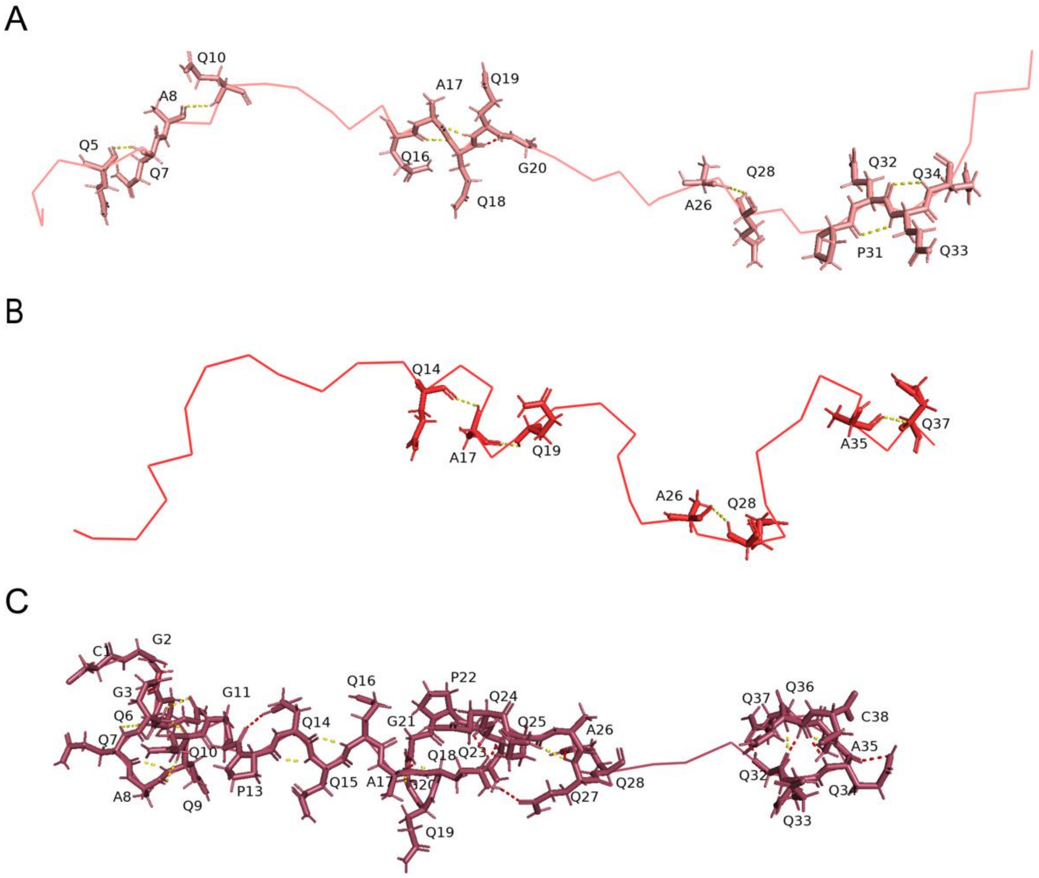

Figure S2. The γ/β-turn structure of DRS in three states. Ribbon representation of the γ/β-turn structure of DRS free in solution (A, salmon), bound with wPDI^{O} (B, tv_red) and wPDI^{R} (C, raspberry). Amino acids involved in structural maintaining hydrogen bonding are shown in sticks. Yellow dotted line represents hydrogen bonds involved in the γ/β-turn structures and red dotted line represents hydrogen bonds not involved.

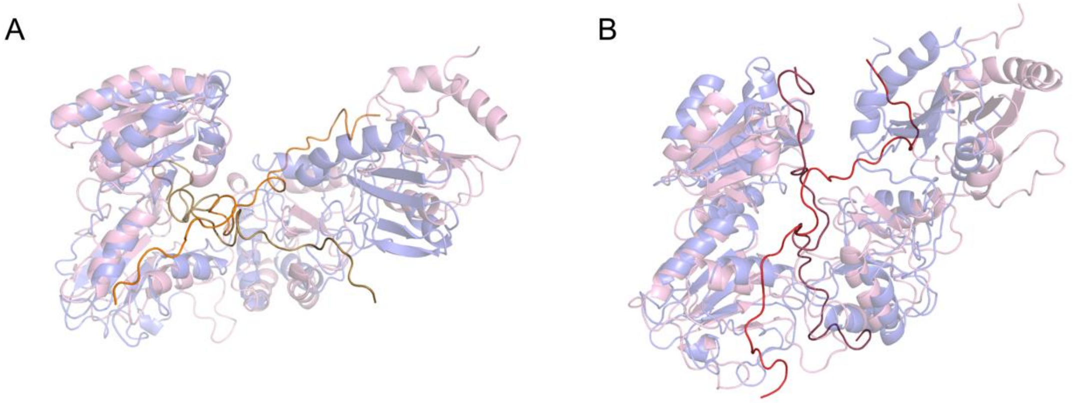

Figure S3. Conformational change of repetitive sequence in the redox cycle of wPDI. A, carton representation of WRS (orange, sand) bound to wPDI^{O} (pink) and wPDI^{R} (slate). B, carton representation of DRS (tv_red, raspberry) bound to wPDI^{O} (pink) and wPDI^{R} (slate).

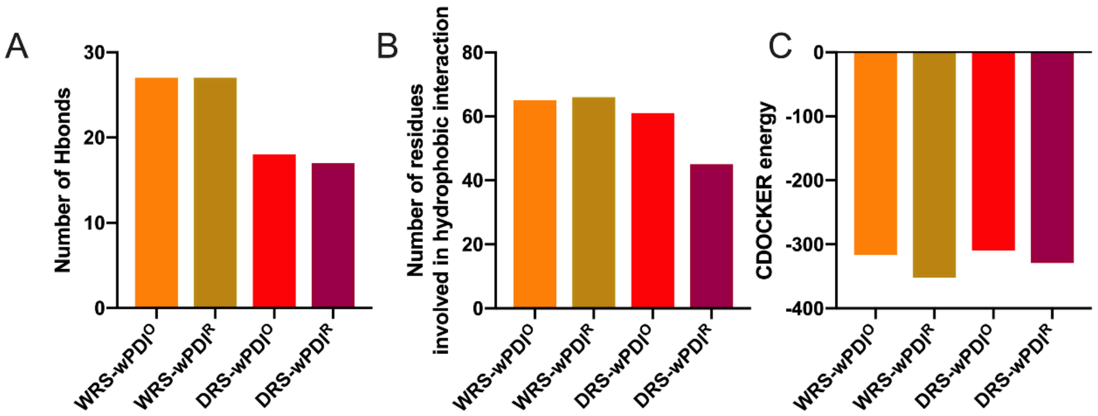

Figure S4. Statistics of interactions between repetitive sequences and wPDI. A, B, and C displays the number of hydrogen bonds, number of amino acid residues involved in the hydrophobic interactions, and CDOCKER energy involved between WRS and DRS interaction with wPDI at different redox states.
